# Supplementary material for: Photoinduced Processes in Lysine-Tryptophan-Lysine Tripeptide with L and D Tryptophan
Source: Int J Mol Sci. 2023 Feb 7;24(4):3331. doi: 10.3390/ijms24043331 (PMC9967182; doi:10.3390/ijms24043331)
Supplement: Supplementary file 1 [file ijms-24-03331-s001.zip › ijms-2159829-supplementary.pdf]

SUPPLEMENTARY MATERIALS

Photoinduced Processes in Lysine-Tryptophan-Lysine Tripeptide with L and D Tryptophan

Aleksandra A. Ageeva [1,2], Roman S. Lukyanov [1,2], Sofia O. Martyanova [1,2], Ilya M. Magin [1], Alexander I. Kruppa [1], Nikolay E. Polyakov [1], Victor F. Plyusnin [1,2], Alexander B. Doktorov [1,*] and Tatyana V. Leshina [1]

[1] V.V. Voevodsky Institute of Chemical Kinetics and Combustion, Siberian Branch of the Russian Academy of Sciences, 3 Institutskaya Str., 630090 Novosibirsk, Russia
[2] Department of Natural Sciences, Department of Physics, Novosibirsk State University, 2 Pirogova Str., 630090 Novosibirsk, Russia
* Correspondence: author: doktorov@kinetics.nsc.ru

UV-visible spectrophotometry

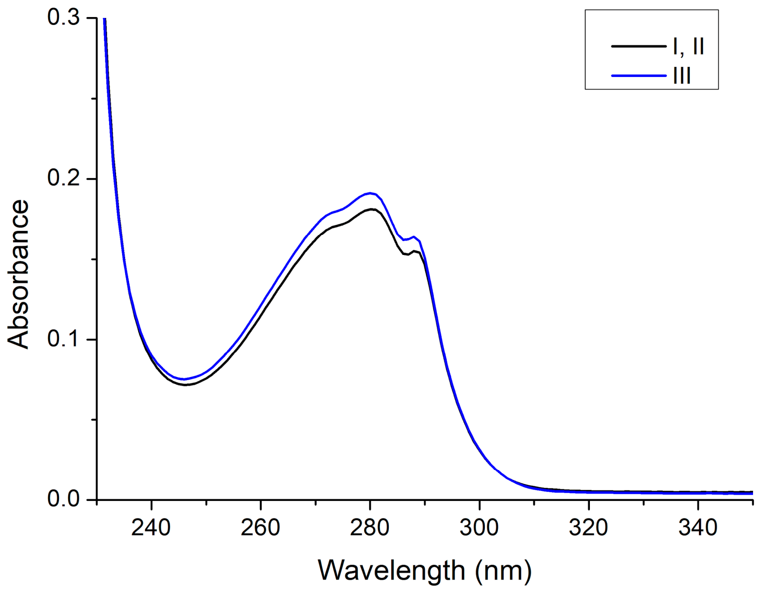

Figure S1. Absorption spectra of peptides **I-III** in water in a 1 cm quartz cuvette, concentration *ca.* $3x10^{-5}$ M.

^{1}H NMR investigation of photolysis products

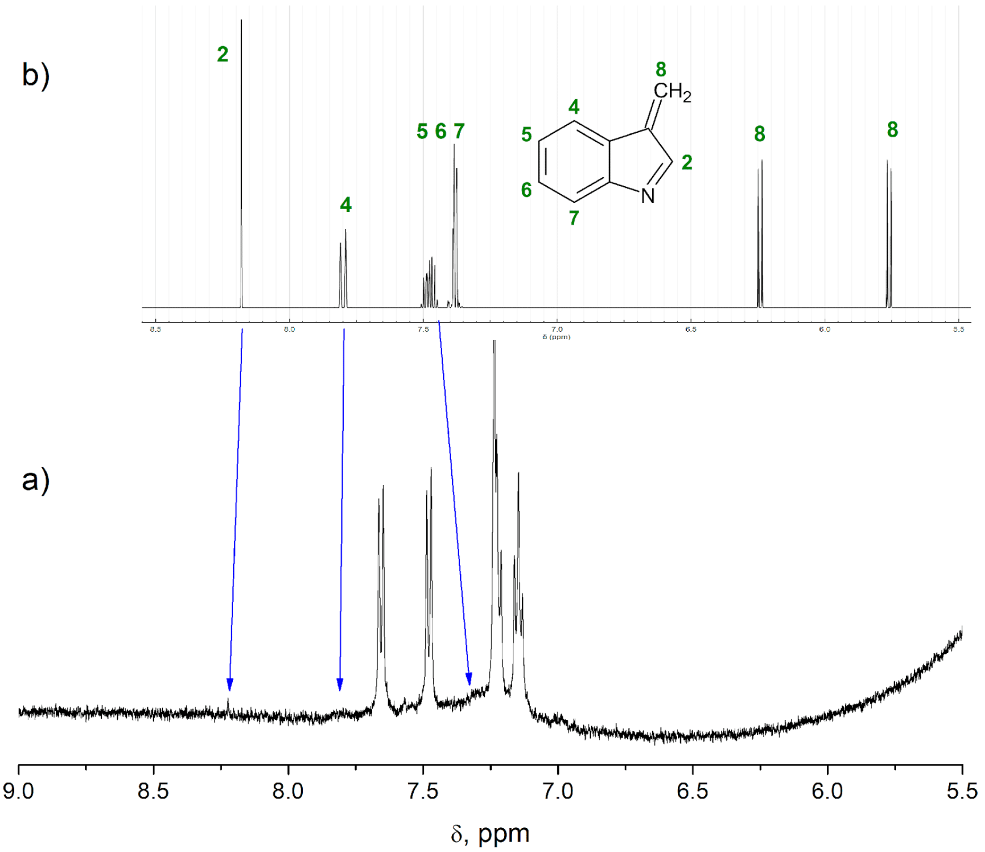

Figure S2. 500 MHz 1H NMR spectra of the reaction mixture after photolysis of 3 mM aqueous solutions of **II** (a) with NMR simulation of 3-methyleneindolenine (b).

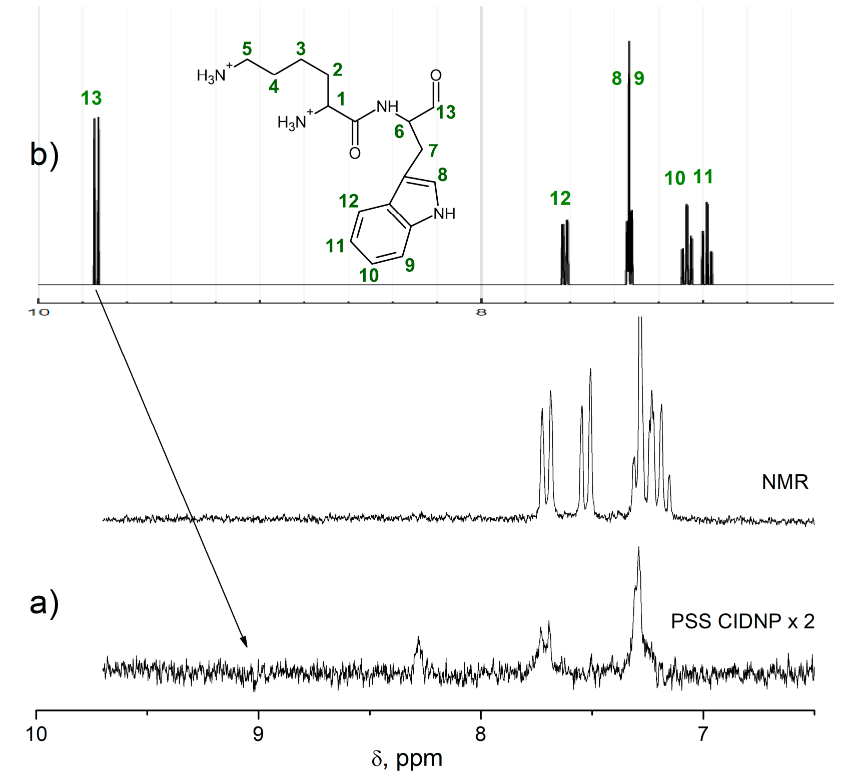

Figure S3. 200 MHz ^{1}H NMR and CIDNP spectra of the aromatic area of the reaction mixture after photolysis of 3 mM aqueous solutions of **II** (a) with NMR simulation of aldehyde (b).

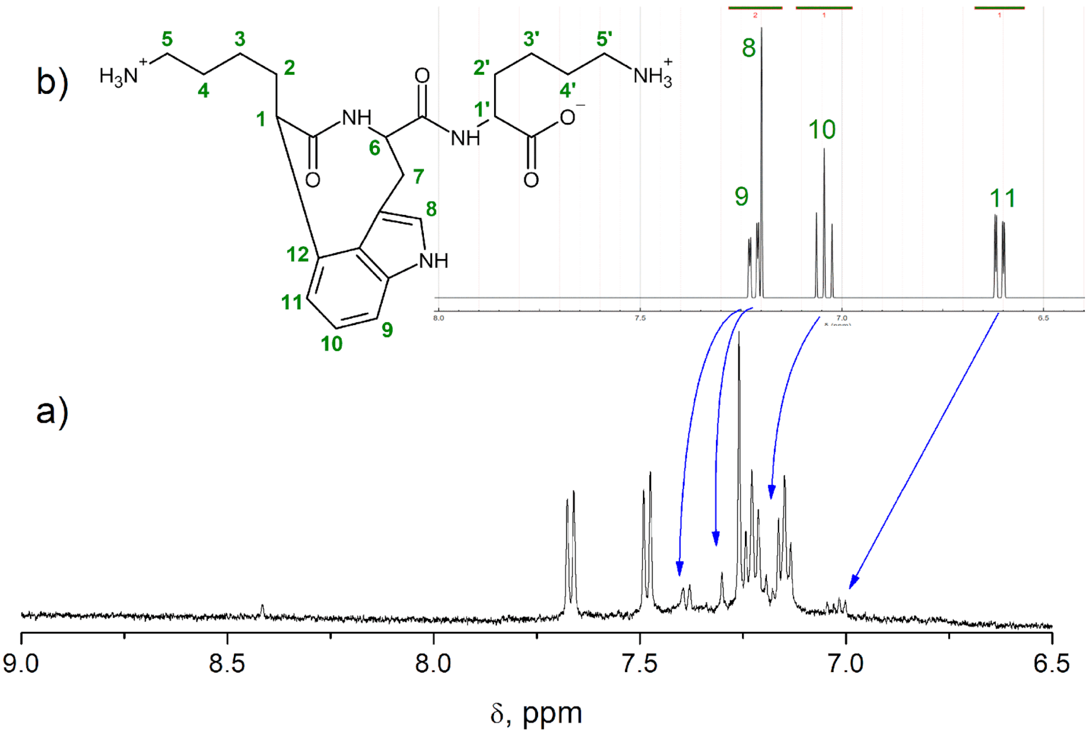

Figure S4. 500 MHz ^{1}H NMR spectra of the reaction mixture after photolysis of 3 mM aqueous solutions of **III** (a) with NMR simulation of the cyclic product (b).

Table S1. Consumption of signal integral intensity under UV irradiation relative to the starting compound **I**.

Proton number	ν(F1) [ppm]	Consumption				
		1 mM	2 mM	2.8 mM	5 mM	7.1 mM
12	7,64	0,18	0,12	0,14	0,07	0,06
9	7,48	0,16	0,12	0,10	0,08	0,08
8,10,11	7,18	0,18	0,10	0,13	0,09	0,06
1, 1'	4,12	0,25	0,13	0,06	0,08	0,02
7	3,29	0,15	0,12	0,14	0,09	0,05
5, 5'	2,89	0,08	0,03	0,03	-0,01	0,01
$CH_3C(O)$-	1,91	0,16	0,10	0,09	0,08	0,06
2, 4, 2',4'	1,63	0,06	0,01	0,03	0,01	0,01
3, 3'	1,21	0,09	0,06	0,05	0,06	0,03

Table S2. Consumption of signal integral intensity under UV irradiation relative to the starting compound **II**.

Proton number	ν(F1) [ppm]	Consumption				
		1 mM	2 mM	3 mM	5,5 mM	8 mM
12	7,72	0,26	0,06	0,18	0,09	0,08
9	7,50	0,26	0,07	0,16	0,08	0,10
8,10,11	7,19	0,28	0,08	0,18	0,09	0,10
1	4,32	0,38	-0,07	0,27	0,03	0,14
1'	4,08			0,26	0,02	0,08
7	3,33	0,52	-0,10	0,37	0,17	0,14
5	2,95	0,23	0,32	0,10	0,00	-0,01
5'	2,70	0,37	0,20	0,15	0,10	0,09
$CH_3C(O)$-	2.00	0,00	0,12	0,05	0,05	0,02
2, 4, 2',4'	1,6044	0,11	-0,03	0,03	0,00	0,01
3, 3'	1,0362	0,36	0,16	0,16	0,08	0,07

Table S3. Consumption of signal integral intensity under UV irradiation relative to the starting compound **III**.

Proton number	ν(F1) [ppm]	Consumption				
		1 mM	2 mM	3 mM	5,5 mM	8 mM
12	7,72	0,19	0,20	0,23	0,20	0,17
9	7,52	0,19	0,14	0,08	0,11	0,08
8,10,11	7,22	0,14	0,08	0,09	0,11	0,07
1	4,11	0,32	0,18	0,25	0,21	0,16
1'	3,88	0,41	0,19	0,33	0,34	0,25
7	3,36	0,33	0,21	0,21	0,26	0,20
5, 5'	2,94	0,15	0,05	0,06	0,08	0,05
CH_3COO-	1,96	-0,03	-0,01	-0,03	-0,02	0,02
2, 4, 2',4'	1,69	0,11	0,06	0,08	0,10	0,06
3	1,38	0,13	-0,04	0,01	0,01	0,02
3'	1,14	0,06	-0,01	0,06	0,11	0,05

CIDNP effects and CIDNP coefficients dependencies

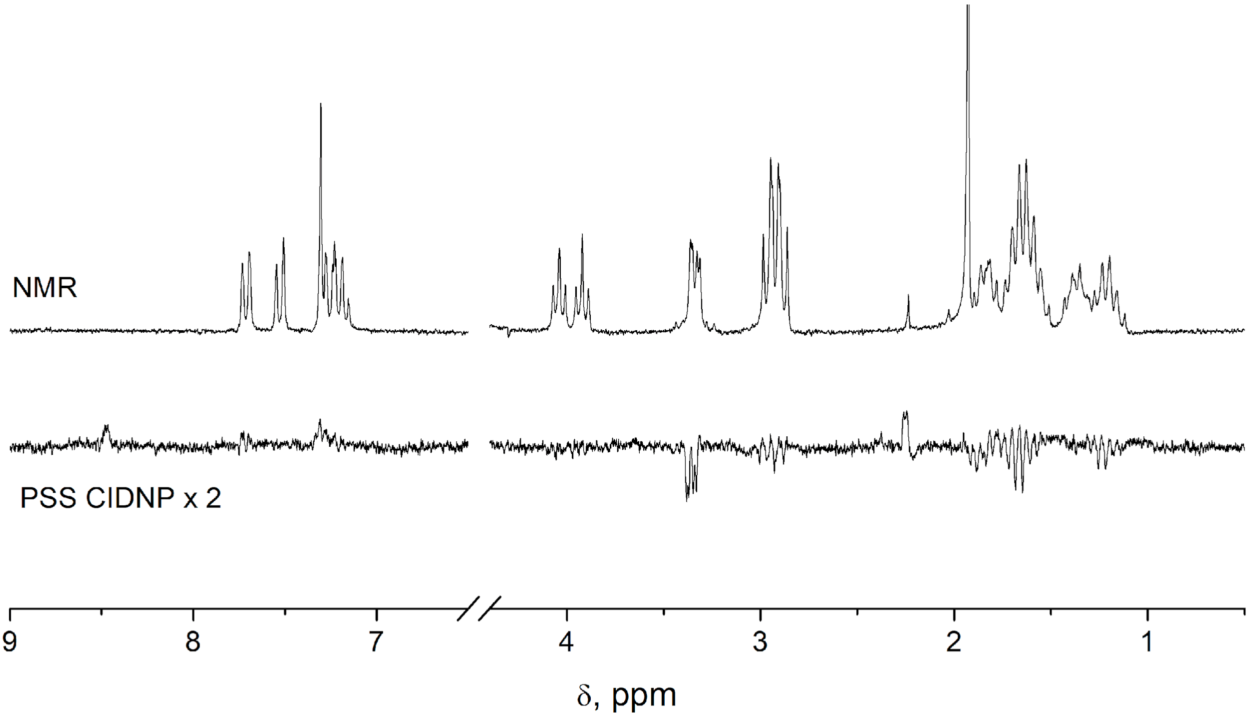

Figure S5. CIDNP effects of 3 mM solution of **III** in water-d2.

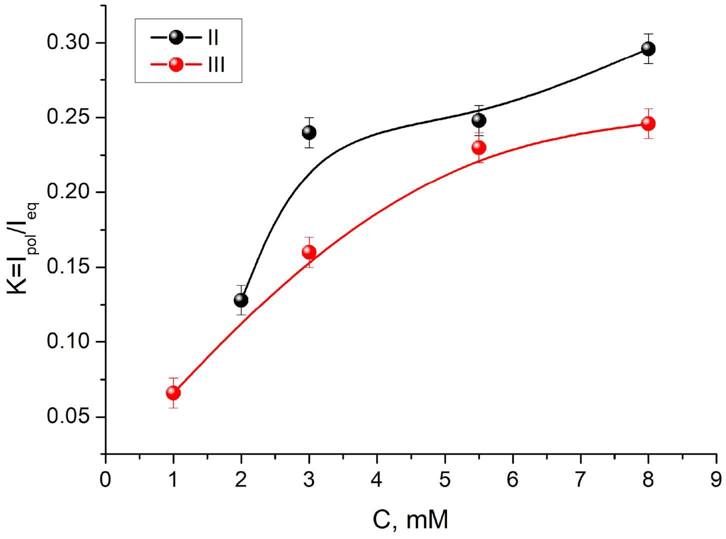

Figure S6. Concentration dependence of the CIDNP coefficients of 3-methyleneindolenine protons in 2 position (8.3-8.4 ppm) for **II** and **III**.

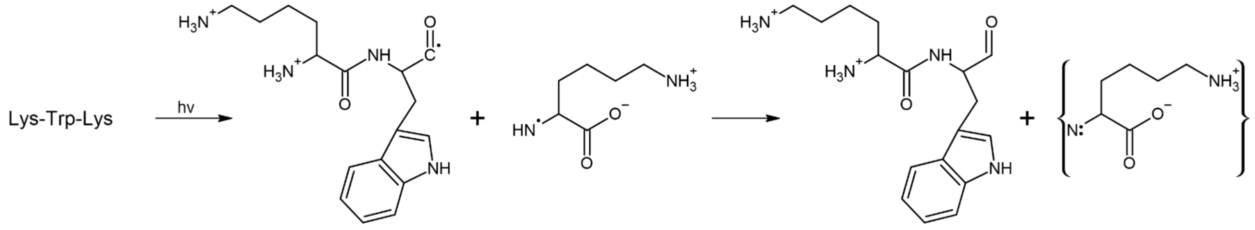

Scheme S1. Possible way of the peptide bond photoinduced cleavage (nitrene reactivity see in [5]).

4. Analysis of CIDNP signs

For high magnetic fields, the sign of the integral CIDNP in the radical pair (RP) of the recombination product is determined by the following parameters: μ,ε, hfi constants and Δg, where μ – the multiplicity of the excited state of reacting molecule which is plus for triplet state and mines for singlet; ε - plus for cage recombination product; hfi signs plus or mines for polarized protons and Δg – a difference of g-factors of the polarized radical and radical partner in RP [1]. To determine the sign of the CIDNP in a pair of radical ions (RIP) or a biradical-zwitterion (BZ), one more parameter is added to the above – γ [2]. It will be a plus for RIP/BZ collective spin singlet state and a minus for triplet. So, the CIDNP sign (S) for initial peptides protons is determined by the following parameters: hfi (aromatic): 12 and 9 H (7.7 ppm; 7.5 ppm) = - 5 G and =-0.5 G; 10H (7.20 ppm)= -4.12 G; 11H (7.25 ppm)= +1.24 G; 8H (7.3 ppm)= -4.21 G; CH_2(7a,b) = +7–10 G, g-factor $NHCO^{-\bullet}$ more than that for $Trp^{+\bullet}$ (2.0031 and 2.0026 [3,4]). Then the protons of the product of back ET in the radical-ions pair (RIP) formed from the triplet excited state of the peptide during singlet recombination have to be polarized as follows: S = μ × ε × hfi × Δg × γ = + × + × - × - × + = A (positive polarization) for 8, 9, 12 aromatic protons; a negative for 11 aromatic proton and 7 CH_2, since the sign of hfi is positive. In the case of the net CIDNP sign (S) of methyl protons (E, emission,1.9 -2.0ppm in Figure 4 of MS) of the amide group, it corresponds to the formation of the CIDNP in the singlet RIP of the tryptophan radical-cation and the radical - anion of [NH C·OCH$_3$]. The difference between the g-factors in this pair is positive for the methyl group, while the hfi is positive. Then, S = μ × ε × hfi × Δg × γ = - × + × + × + × + = E. In the case of 3-methyleneindolenine, protons in 2 position, positive polarization is associated with the following parameters: S = μ × ε × hfi × Δg ×γ = + × + × - × - ×+ = A (scheme X, Δg < 0, hfi of CH in the 2 position is negative).

5. Calculations of the spin density distribution

Structure optimization of peptide **I** radical-anion and calculation of spin density distribution in this radical were performed by density functional theory (DFT) calculations in vacuo, using PBE functional [6] and the quantum-chemical program PRIRODA [7,8] with L1 basis [9].

Table S4. DFT calculation of HFI constants of peptide I radical-anion.

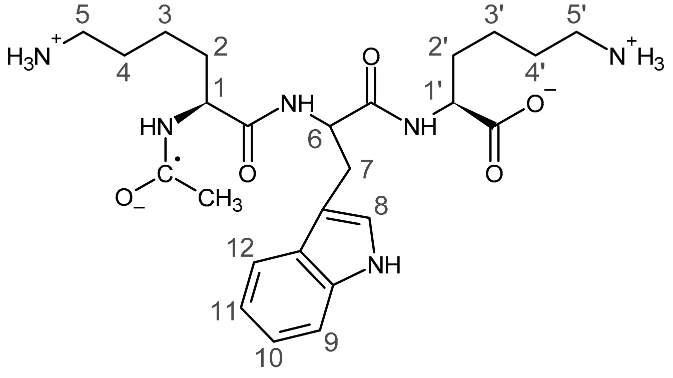

**Proton position**	**HFI, G**
1	3.3
6	5.4
$NHCOCH_3$	0<br>3.2<br>2.8

References

1. Kaptein, R. (1971). Simple rules for chemically induced dynamic nuclear polarization. Journal of the Chemical Society D: Chemical Communications, (14), 732. doi:10.1039/c29710000732

2. Closs, G. L., & Czeropski, M. S. (1977). Amendment of the CIDNP phase rules. Radical pairs leading to triplet states. Journal of the American Chemical Society, 99(18), 6127–6128. doi:10.1021/ja00460a061

3. Babenko, S. V., Kuznetsova, P. S., Polyakov, N. E., Kruppa, A. I., & Leshina, T. V. (2020). New insights into the nature of short-lived paramagnetic intermediates of ketoprofen. Photo-CIDNP study. Journal of Photochemistry and Photobiology A: Chemistry, 112383.

4. Saprygina, N. N., Morozova, O. B., Gritsan, N. P., Fedorova, O. S., & Yurkovskaya, A. V. (2011). 1H CIDNP study of the kinetics and mechanism of the reversible photoinduced oxidation of tryptophyl-tryptophan dipeptide in aqueous solutions. Russian Chemical Bulletin, 60(12), 2579–2587.

5. C. Wentrup, (1984). *Reactive Intermediates*. New York: Wiley.

6. Perdew, J.P.; Burke, K.; Ernzerhof, M. Generalized Gradient Approximation Made Simple. *Phys. Rev.* **1996**, *77*, 3865–3868.

7. Laikov, D.N. Fast Evaluation of Density Functional Exchange-Correlation Terms Using the Expansion of the Electron Density in Auxiliary Basis Sets. *Chem. Phys. Lett.* **1997**, *281*, 151–156.

8. Laikov, D.N.; Ustynyuk, Y.A. PRIRODA-04: A Quantum-Chemical Program Suite. New Possibilities in the Study of Molecular Systems with the Application of Parallel Computing. *Russ. Chem. Bull.* **2005**, *54*, 820–826.

9. Laikov, D.N. A New Class of Atomic Basis Functions for Accurate Electronic Structure Calculations of Molecules. *Chem. Phys. Lett.* **2005**, *416*, 116–120.
